# RETRACTION: Effect of Prophylactic Central Neck Dissection Following Total Thyroidectomy on Surgical Site Wound Infection, Hematoma, and Haemorrhage in Subjects With Clinically Node‐Negative Papillary Thyroid Carcinoma: A Meta‐Analysis

**DOI:** 10.1111/iwj.70461

**Published:** 2025-04-02

## Abstract

**RETRACTION:** JinL. , LiuL. , WangJ. , and ZhangL. , “Effect of Prophylactic Central Neck Dissection Following Total Thyroidectomy on Surgical Site Wound Infection, Hematoma, and Haemorrhage in Subjects With Clinically Node‐Negative Papillary Thyroid Carcinoma: A Meta‐Analysis,” International Wound Journal 20, no. 2 (2023): 251–260, 10.1111/iwj.13867.35702946 PMC9885457

The above article, published online on 15 June 2022, in Wiley Online Library (http://onlinelibrary.wiley.com/), has been retracted by agreement between the journal Editor in Chief, Professor Keith Harding; and John Wiley & Sons Ltd. Following an investigation by the publisher, all parties have concluded that this article was accepted solely on the basis of a compromised peer review process. The editors have therefore decided to retract the article. The authors did not respond to our notice regarding the retraction.

**RETRACTION:** L. Jin , L. Liu , J. Wang , and L. Zhang , “Effect of Prophylactic Central Neck Dissection Following Total Thyroidectomy on Surgical Site Wound Infection, Hematoma, and Haemorrhage in Subjects With Clinically Node‐Negative Papillary Thyroid Carcinoma: A Meta‐Analysis,” International Wound Journal 20, no. 2 (2023): 251–260, 10.1111/iwj.13867.35702946 PMC9885457

The above article, published online on 15 June 2022, in Wiley Online Library (http://onlinelibrary.wiley.com/), has been retracted by agreement between the journal Editor in Chief, Professor Keith Harding; and John Wiley & Sons Ltd. Following an investigation by the publisher, all parties have concluded that this article was accepted solely on the basis of a compromised peer review process. The editors have therefore decided to retract the article. The authors did not respond to our notice regarding the retraction.

